# Effects of Exercise Frequency with Complex Contrast Training on Measures of Physical Fitness in Active Adult Males

**DOI:** 10.3390/sports11010011

**Published:** 2023-01-05

**Authors:** Gopal Kumar, Vivek Pandey, Rohit K. Thapa, Anthony Weldon, Urs Granacher, Rodrigo Ramirez-Campillo

**Affiliations:** 1 Department of Exercise Physiology, Lakshmibai National Institute of Physical Education, Gwalior 474002, India; 2 School of Physical Education and Sports, Rashtriya Raksha University, Gandhinagar 382305, India; 3 Centre for Life and Sport Sciences (CLaSS), Faculty of Health, Education and Life Sciences, Birmingham City University, Birmingham B15 3TN, UK; 4 Department of Sport and Sport Science, Exercise and Human Movement Science, University of Freiburg, 79102 Freiburg, Germany; 5 Exercise and Rehabilitation Sciences Institute, School of Physical Therapy, Faculty of Rehabilitation Sciences, Universidad Andres Bello, Santiago 7591538, Chile

**Keywords:** plyometric exercise, human physical conditioning, resistance training, muscle strength, musculoskeletal and neural physiological phenomena, musculoskeletal physiological phenomena, exercise, sports science, sports medicine, athletic performance

## Abstract

Complex contrast training (CCT) is an exercise modality that utilizes both high-load resistance activity and low-load plyometric activity in a set-by-set fashion within a single exercise session. Such a combination of exercises targets multiple aspects of the force–velocity curve and may thus lead to improvement of various components of physical fitness. However, no previous study has attempted to compare the effects of load-equated two vs. three CCT sessions per week on measures of physical fitness. Forty-five male participants aged 21.4 ± 2.0 years were randomly assigned to either two weekly CCT sessions (CCT-2; n = 15), three weekly CCT sessions (CCT-3; n = 15), or an active control group (CG; n = 15). Selected measures of physical fitness were assessed pre- and post-six weeks of training. The tests included the assessment of 15 and 30 m linear sprint speeds, upper (medicine ball throw) and lower limb muscle power (standing long jump and countermovement jump with arm thrust), muscle strength (isokinetic peak knee extensor/flexor torque), and change-of-direction speed (modified agility T-test (MAT)). Significant group–time interactions were observed for all dependent variables (all *p* < 0.001, ɳ_p_^2^ = 0.51–0.78) using ANOVA. Post hoc tests indicated significant performance improvements for the CCT-2 and CCT3 groups for all dependent variables (Hedge’s g = 0.28–3.26, %Δ = 2.4–16.7), including the 15 and 30 m linear sprint speeds (*p* < 0.001), medicine ball throw (*p* < 0.001), standing long jump (*p* < 0.001), countermovement jump with arm thrust (*p <* 0.001), right leg knee extensor (*p* < 0.001) and flexor peak torque (*p* < 0.001), left leg knee extensor (*p <* 0.001) and flexor peak torque (*p <* 0.001), and change-of-direction speed (*p* < 0.001). The CCT-3 group showed greater improvements in MAT compared to the CCT-2 group (g = 3.26 vs. 0.70, *p* < 0.001). In conclusion, compared to active controls, the load-equated CCT-2 and CCT-3 programs provided similar effects on measures of physical fitness in active adult males. However, an athlete’s goal is to improve their MAT score, the CCT-3 program may elicit greater improvements compared with the CCT-2 program.

## 1. Introduction

Sufficient levels of physical fitness (e.g., speed, power, strength, and agility) are important prerequisites for the performance of sports-specific motor skills and for the promotion of motor skill learning [1,2]. Although different resistance training methods have been proposed for the development of health- and skill-related physical fitness [3,4,5], heavy-load resistance training and plyometric training are among the most studied [6,7]. These training methods induce different physiological adaptations as heavy-load resistance training is strength-focused and plyometric training is velocity-focused [8]. Both training methods have the potential to improve maximal strength and muscle power with varying magnitudes through neural (e.g., motor unit activation/recruitment and synchronization), morphological (e.g., cross-sectional area), and cellular, as well as metabolic, adaptations (e.g., fiber-type composition) [6,9]. These adaptations underpin the developments deemed necessary for physical fitness improvement, such as vertical and horizontal jump performance, linear sprint speed, and/or change-of-direction speed [10].

We note that the combination of heavy load resistance exercises with plyometric exercises within a single training session may induce additional improvements in physical fitness that may further enhance physical fitness [8]. The combination of these two exercise regimes compared with single mode exercise could induce adaptation along the force–velocity continuum when compared to exercise protocols using either only heavy load resistance or plyometric training [11]. Complex contrast training (CCT) is a combined exercise type that involves the alternate application of heavy-load resistance exercise with low-load plyometric exercise within one exercise session in a set-by-set fashion [8,12,13]. CCT-related physical fitness improvements include enhanced 15 m linear sprint speed [14], maximal strength (e.g., full squat, leg press, box squat, and lower limbs isokinetic peak torque) [14,15,16], and muscle power (e.g., countermovement jump height (CMJ) and squat jump) [14,15,16]. Furthermore, there is evidence of CCT-induced physical fitness improvements across various populations, including soccer athletes, handball athletes, and physical education students with or without previous strength training experience [12,13,14,15,16,17]. The additional benefits of CCT compared with single-mode exercise protocols may be related to the utilization of the post-activation performance enhancement phenomenon [18]. Indeed, in accordance with previous studies [18,19], higher-load CCT activity may induce greater motor unit recruitment and potentiate subsequent lower-load activity.

Furthermore, with CCT, the exercise programming variables such as intensity (e.g., percentage of the one-repetition maximum [1-RM]) and total load (e.g., total sets/repetitions) are well-researched [8]. However, according to the FIIT principle (i.e., frequency, intensity, time, and type), exercise frequency is another important programming parameter that requires further investigation with CCT. More specifically, there is a need to clarify the effects of different CCT frequencies under load-equated conditions on the physical fitness of healthy participants. There are several theoretical advantages of increased resistance or plyometric training frequency with equated total load. For example, increased protein synthesis in response to resistance training may last for 24–48 h in untrained individuals [20] and 24 h in trained individuals [21]. Consequently, higher training frequencies may provide more time with a net positive protein balance, thus enhancing muscular adaptations [22]. Similarly, greater weekly plyometric training frequency may favor bone mass accretion [23]. Furthermore, distributing the same weekly load across higher frequencies (i.e., several days) may reduce fatigue during single-exercise sessions [22] and recovery duration between sessions [24]. Lastly, more frequent exercise stimuli during a weekly training schedule may optimize motor skill learning [25]. However, there are contradictory findings reported in the literature. One study [16] reported a 7.7% CMJ improvement after six weeks of CCT with two weekly sessions, while another study [15] reported a −1.4% CMJ reduction in CMJ height for three weekly CCT sessions. Moreover, based on a meta-analysis on resistance training frequency, higher and lower training frequencies (i.e., ≥4, 3, 2, and 1 session/week) are similarly effective for improving muscle strength given that total training load is equated [26]. Similarly, in a recent review by Ramirez-Campillo et al. [27], there were no effects reported for load-equated plyometric jump training frequency on soccer players’ physical fitness. 

Of note, resistance training, plyometric jump training, and CCT may target the force–velocity continuum differently (e.g., either force or velocity and combined force–velocity). Therefore, extrapolations from single-mode resistance training or plyometric jump training to CCT may be inappropriate. Accordingly, well-designed studies (e.g., load-equated) that compare the effects of different weekly CCT frequencies are required. Therefore, this study aimed to contrast the effects of two vs. three load-equated weekly CCT training sessions on selected measures of physical fitness in active adult males, including 15 and 30 m linear sprint speeds, upper (medicine ball throw (MBT)) and lower limbs muscle power (standing long jump (SLJ), countermovement jump with arm thrust (CMJA)), muscle strength (isokinetic knee flexor/extensor peak torque), and change-of-direction speed (modified agility T-test (MAT)). Based on the available literature [22,25], we hypothesized that load-equated training interventions with three weekly sessions would induce greater improvements compared to two weekly sessions on measures of physical fitness. We further hypothesized that CCT interventions would induce greater improvements compared to an active control group (CG). Furthermore, considering the novelty of the study, the results may be useful to practitioners for making evidence-based decisions regarding choosing a weekly CCT frequency for the optimization of physical fitness.

## 2. Materials and Methods

The study was designed according to the international guidelines for quality-based randomized controlled trials [28,29,30]. 

### 2.1. Experimental Design

A two (within-subject; pre- and post-intervention measurements) by three (between-subjects; CCT-2, CCT-3, and CG) randomized study design was used to compare the effects of different exercise frequencies on linear sprint speed, muscle power and strength, and change-of-direction speed. Pre- and post-intervention measurements were performed at similar times during the day for all participants, with linear sprints, MBT, SLJ, CMJA, and MAT conducted on day one (6:00–8:00 AM) and isokinetic testing conducted 24–72 h after day one (2:30–5:30 PM). The sequence of the testing order was the same for all participants and tests (pre- and post-intervention). Upon arrival in the laboratory, the participants underwent a 10-min general warm-up. For outdoor assessments, temperature, humidity, and wind velocity ranged from 28–31 °C, 15–65 %, and 0–10.8 km.h^−1^, respectively, during the baseline and post-intervention assessments. 

**Figure 1:**
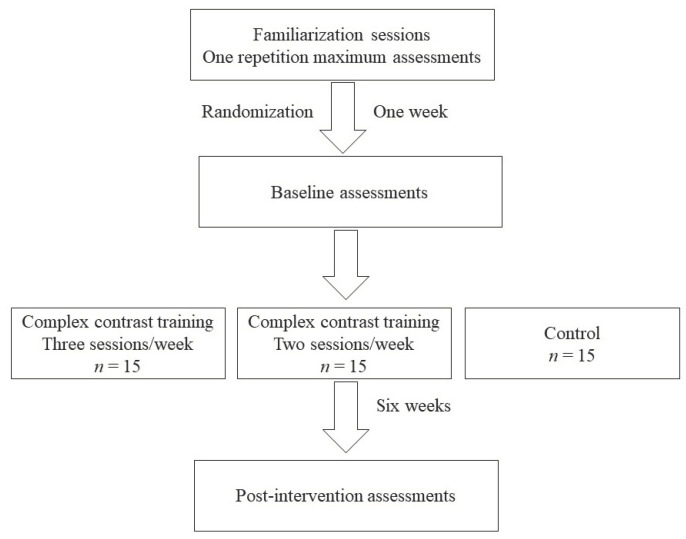
Schematic representation of the study.

Participants performed three familiarization sessions including CCT exercises and two familiarization sessions for the testing procedures that were undertaken one and two weeks before the baseline testing. Demographic and anthropometric data were collected and 1-RM tests were performed at least one week before the baseline testing during the familiarization sessions, and these included squats, barbell lunges, Romanian deadlifts, and bench presses. The results from the 1-RM testing were used to program the exercise interventions. The participants were asked to (i) refrain from strenuous activity 24 h before testing, (ii) eat and drink habitually, and (iii) refrain from consuming caffeine three hours before testing. A schematic representation of the study is provided in Figure 1.

### 2.2. Participants

The required sample size for this study was estimated using statistical software (G*power; University of Düsseldorf, Düsseldorf, Germany). The following variables were included in the a priori power analysis: study design with three groups; two measurements; an alpha error of <0.05; a non-sphericity correction of 1; a correlation between repeated measures of 0.5; a desired power (1-ß error) of 0.80; and an effect size (f) of 0.27, all based on prior research investigating the effects of six-weeks of CCT on amateur soccer players’ 30 m linear sprint speed performance [31].

The results of the a priori power analysis indicated that a minimum of 13 participants was required for each group to achieve statistical significance for the main outcome of the study (linear sprint speed (i.e., 30 m linear sprint speed)). Accordingly, 45 male participants were recruited for this study, with a slightly higher number of participants than recommended in case any participants dropped out (e.g., injury not related to the intervention). The eligibility criteria for this study required participants who were: (1) university students, (2) actively participating in sports (e.g., basketball) as part of their course curriculum or in other forms of physical activity (e.g., running) for a minimum duration of five hour per week, (3) having a minimum of one year of resistance training/testing experience and able to perform all the exercises included in the study’s training protocol, and (4) free from lower limb injuries for at least six months before this study. Participants were randomly assigned (using the randomization tool www.randomizer.org) to either two weekly CCT sessions (CCT-2; n = 15), three weekly sessions (CCT-3, n = 15), or an active control group (CG, n = 15). The number of total repetitions and load used in the experimental groups was equated across the groups. The participants within each group possessed similar demographics and anthropometrics, except for body mass (*p* = 0.028) (Table 1). The potential risks and benefits of this study were explained to the participants before the study. Thereafter, written informed consent was obtained from all individuals. The local ethics committee of the university approved this study, and the study was conducted according to the latest version of the Declaration of Helsinki.

### 2.3. Load Measurement for Training Prescription

Before the start of the training intervention, 1-RM assessments were conducted according to the methods outlined in a previous study [31]. Before testing, a 10-min general warm-up was conducted that included jogging, dynamic stretching, and body mass exercises (e.g., freehand squat, walking lunges, and push-ups). A short, specific warm-up consisting of 5–10 repetitions with a load of 40–60%, as well as 3–5 repetitions at 60–80% of the estimated 1-RM, was performed. Thereafter, the load was gradually increased in increments of 10 kg or less to achieve the 1-RM within a maximum of five attempts. The rest period between 1-RM attempts was four minutes. The absolute and relative 1-RMs obtained for the squat, lunge, Romanian deadlift, and bench press were similar at baseline for the CCT-2 and CCT-3 groups (Table 2). No 1-RM data were collected for the active CG.

### 2.4. Training Intervention

Six weeks of CCT interventions were considered [16], and biomechanically similar exercises [8] were selected for the contrast pairs used during CCT, as follows: (i) squat with CMJ, (ii) Romanian deadlift with kettlebell swings, (iii) lunges with barbell high knees, and (iv) bench press with plyometric push-ups. The CMJ and plyometric push-ups were performed without external resistance, kettle bell swings were performed with 10–20 kg, and barbell high knees were performed with a 20 kg Olympic barbell. The participants were asked to perform both the high-load and low-load activities with the intention (i.e., effort) of attaining maximal velocity. The low-load activity was performed immediately after the high-load activity with no specified rest period. However, one minute of recovery was allowed between consecutive sets, two minutes were allowed between contrast pairs (i.e., between squat and CMJ), and ≥48 h were allowed between sessions. A total of 12 and 18 CCT sessions were completed by the CCT-2 and CCT-3 groups, respectively. During the intervention period, the participants in the active CG were involved in regular physical activity classes and non-competitive sports similar to the CCT-2 and CCT-3 groups. More details on the training protocol used across the six-week intervention period are presented in Table 3.

### 2.5. Physical Fitness Tests

All the measurements were conducted by the same independent assessors pre- and post-intervention. In addition, the assessors were blinded to the group allocation of the participants.

#### 2.5.1. Speed

Linear sprint speed protocols were adapted from the methods outlined in a previous study [32] and conducted on an outdoor synthetic track. Participants were instructed to stand behind a start line with a self-selected leg forward and start only after the command of the assessor. Two independent assistants who were not part of this study were recruited as timekeepers (the between-timekeepers interclass correlation coefficients (ICCs) were 0.99 for both the 15 m and 30 m distances) and assigned to record the timing of each trial using a hand stopwatch (Casio S053 HF-70W-1DF, Casio Computer Co., Ltd., Tokyo, Japan). The times recorded by the two timekeepers were averaged for the analysis. Three trials were conducted for the 15 m and 30 m linear sprint tests with a one-minute recovery between trials, and the fastest trial was selected for further analysis. The ICC for test–retest was 0.86 (95% confidence interval (CI): 0.75–0.92) for the 15 m sprint test and 0.87 (95% CI: 0.77–0.93) for the 30 m sprint test.

#### 2.5.2. Upper Limb Muscle Power

For the performance of the MBT, participants stood on the start line with their feet shoulder-width apart. Thereafter, participants threw a three kg medicine ball backward overhead. The distance between the start line and where the ball first contacted the floor was measured using a standard measuring tape. The test was performed as described previously by Stockbrugger and Haennel [33]. Two trials were conducted, and the furthest throw was selected for analysis. The ICC for test–retest was 0.97 (95% CI: 0.95–0.98).

#### 2.5.3. Lower Limb Muscle Power

The SLJ protocol was adapted from methods outlined in a previous study [34] and conducted on a synthetic outdoor track. Participants stood behind a start line with their feet slightly apart and were instructed to swing their arms and perform a countermovement to a self-selected depth before taking off and landing with both legs. Verbal encouragement was provided to jump as far as possible. The measurement was recorded from the start line to the nearest point of contact on the landing (i.e., back of the nearest heel). Three jumps were performed with one minute of rest between jumps, and the longest jump was selected for analysis. The ICC for test–retest was 0.95 (95% CI: 0.91–0.97).

An inertial moment sensor (BTS G-walk, Italy) was used to measure the countermovement jump performance with arm thrust (CMJA). A pilot study reported the sensor to be valid and reliable (concurrent to MyJump 2 (ICC = 0.96, r = 0.973, mean difference = 0.2 ± 1.3, and paired t-test *p* = 0.550)) for measuring the CMJ performance. The sensor was placed on the lower back using a belt with the center of the device at the fifth lumbar vertebrae. Participants stood with their feet slightly apart and were instructed to swing their arms and perform a countermovement to a self-selected depth before taking off and landing with both legs. Knee flexion was not permitted during the flight phase of the jump. Three trials were performed with one minute of rest between jumps, and the best trial was selected for analysis. The ICC for test–retest was 0.96 (95% CI: 0.93–0.98).

#### 2.5.4. Change-of-Direction Speed

The MAT was used to determine speed with directional changes, including forward sprinting, left and right shuffling, and backward running. The protocol was adapted from methods outlined in a previous study [19]. Two independent assistants who were not part of this study were recruited as timekeepers (between-timekeepers ICC was 0.98) and assigned to record the timing of each trial using a hand stopwatch (Casio S053 HF-70W-1DF, Casio Computer Co., Ltd., Tokyo, Japan). The average time recorded by both timekeepers was used for analysis. Three trials were performed with one minute of rest between trials, and the fastest trial was selected for analysis. The ICC for test–retest was 0.97 (95% CI: 0.95–0.98).

#### 2.5.5. Muscle Strength

The isokinetic muscle strength tests were conducted on a HUMAC NORM isokinetic dynamometer (Computer Sports Medicine Inc., Stoughton, MA, USA). A 10-min warm-up was completed before the test, and it included jogging and dynamic stretching of the lower limbs. Thereafter, the participants sat on the machine’s chair, with the axis of rotation of the dynamometer arm aligned with the axis of rotation of the knee. The ‘Knee Extension/Flexion’ test was selected to be performed with the isokinetic ‘CONC/CONC’ mode; therefore, all knee extension and flexion movements involved concentric actions. The right side was always selected first across all testing sessions. The test protocol included a set of six repetitions at 60°/seconds. Two sets were completed for each leg with one minute of rest between the sets. Verbal instructions were provided to push and pull as hard and fast as possible throughout the full range of motion. Furthermore, the screen was positioned so that participants could see the real-time feedback on their effort. Two sets were performed, and the highest peak torque value obtained was selected for analysis. The ICC for test–retest was 0.99 (95% CI: 0.98–0.99) for right knee extension, 0.98 (95% CI: 0.97–0.99) for right knee flexion, 0.99 (95% CI: 0.98–0.99) for left knee extension, and 0.98 (95% CI: 0.97–0.99) for left knee flexion.

### 2.6. Statistical Analysis

Following an intention-to-treat approach, the analyses were conducted using IBM SPSS version 20.0.0 (IBM, New York, NY, USA). Data normality was tested and confirmed using the Shapiro–Wilk test. Accordingly, the data are presented as means and standard deviations. A two (time: pre- and post-intervention tests) by three (group: CCT-2, CCT-3, and CG) mixed ANOVA for repeated measures was used to analyze the exercise-specific effects. Furthermore, in case of significant group–time interactions, Bonferroni-adjusted paired (within-group) and independent (between-group comparisons at post-intervention) t-tests were used for the post hoc analyses. In the case of significant between-group baseline differences, an ANCOVA with baseline values as covariates was further used to verify the intervention effects. Bonferroni-adjusted post hoc tests were applied. Percentage change scores were calculated for each variable in each group using the following equation in Microsoft excel: ((mean_post_ − mean_pre_)/mean_pre_) × 100. Effects sizes (ES) in the form of partial eta squared (ɳ_p_^2^) were used from the ANOVA output. A Hedge’s *g* derived from the paired t-test was calculated to assess the group-specific changes between the pre- and post-intervention measurements. The magnitude of effects for the ɳ_p_^2^ was interpreted as small (<0.06), moderate (≥0.06–0.13), and large (≥0.14) [35], while the Hedge’s *g* was interpreted as trivial (<0.2), small (0.2–0.6), moderate (>0.6–1.2), large (>1.2–2.0), very large (>2.0–4.0), and extremely large (>4.0) [36]. The ICC between the trials and assessors was interpreted having as poor (<0.5), moderate (0.5–0.75), good (0.75–0.9), and excellent (>0.9) reliability based on the lower bound of the 95% confidence interval (CI; ICC_95%CI lower bound_) [37]. Statistical significance was set at *p* ≤ 0.05.

## 3. Results

**Figure 2:**
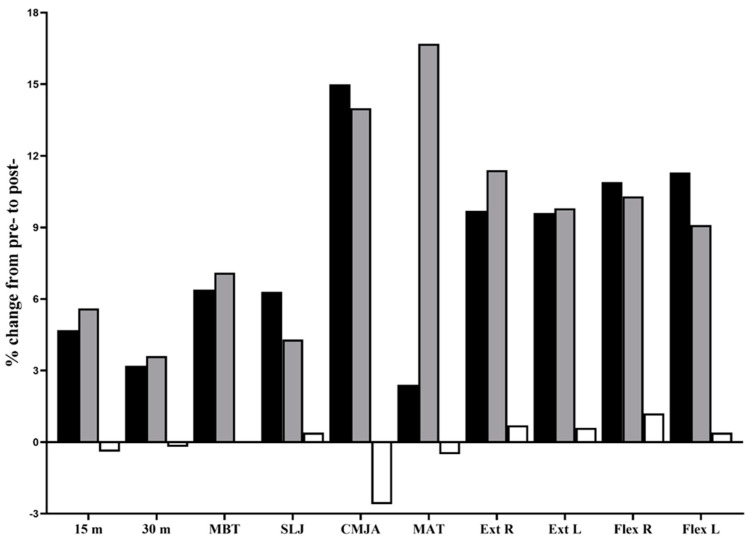
Relative (%) change in dependent variables between the pre- and post-training intervention tests for the complex contrast training two-session group (CCT-2; black bars), three-session group (CCT-3; grey bars), and control group (CG; white bars). For all parameters, significant group–time interactions were noted. The Hedge’s g ranged from 0.43 to 1.61 for the CCT-2 group and from 0.28 to 3.26 for the CCT-3 group.

All participants received the treatments as allocated. No training- or test-related injuries were observed. Adherence to training was 100%. The results for all dependent variables of the main analysis are presented in Table 4, with a graphical representation of the pre- and post-intervention changes (deltas) shown in Figure 2. No baseline between-group differences (one-way ANOVA, *p* = 0.061–0.864) were observed for the measures of linear sprint speeds, SLJ, and isokinetic peak knee flexor/extensor torque. However, significant between-group (CCT-3 vs. CCT-2) baseline differences were found for the upper/lower limb muscle power (MBT and CMJA) and change-of-direction speed (MAT), with significantly lower values in the CCT-3 group (*p* = <0.001–0.01). 

Significant group–time interactions were observed for all dependent variables (all *p* < 0.001, ɳ_p_^2^ = 0.51–0.78), with post hoc tests revealing differences in all variables that favored the CCT-2 and CCT-3 groups compared to the CG group (all *p* < 0.05). The MATs at the post- intervention tests favored (*p* < 0.001) the CCT-3 group (g = 3.26) compared to the CCT-2 group (g = 0.70). Within-group analyses revealed improvements in all dependent variables in the CCT-2 (all *p* < 0.001; g = 0.43–1.61; %Δ = 2.4–15.0) and CCT-3 groups (all *p* < 0.001; g = 0.28–3.26; %Δ = 3.6–16.7), but not in the CG group (*p* = 0.104–0.942; g = 0.00–0.20; %Δ = 0–2.6).

Note: negative bars denote detrimental changes in physical fitness performance. 15 m and 30 m, linear sprint distances; CMJA, countermovement jump with arm thrust; Ext, maximal knee extension isokinetic torque; Flex, maximal knee flexion isokinetic torque; L, left; MAT, modified agility T-test; MBT, medicine ball throw; R, right; SLJ, standing long jump distance. 

## 4. Discussion

Compared to the active controls, six weeks of CCT was effective for improving the selected measures of physical fitness in active adults. However, the load-equated CCT-3 and CCT-2 groups showed similar improvements for most measures of physical fitness, including the 15 m and 30 m linear sprint speeds, muscle strength, and power. Of note, change-of-direction speed improved more in the CCT-3 group compared to the CCT-2 group. 

The primary findings of this study are in line with previous CCT research in similar populations [14,16]. For example, a previous study [14] reported improvements in linear sprint speed (e.g., 15 m) and vertical jump height (e.g., CMJ and squat jumps) after an eight-week CCT intervention with active undergraduate students. Likewise, six-week CCT programs have been reported to improve knee extension and flexion at 60°/s and CMJ height in recreationally trained male and female undergraduate students [16]. The improvements observed in the CCT groups may be attributed to specific neuromuscular adaptations such as improved stretch–shortening cycle, increased motor unit recruitment, firing frequency, intra- and inter-muscular coordination, and morphological changes that support muscle force generation [8,12,13]. Moreover, CCT incorporates both high-load, low-velocity (e.g., heavy squat) and low-load, high-velocity (e.g., CMJ) exercises, helping induce specific adaptations and optimizing the force–velocity relationship [8], whereas programming heavy resistance exercise or plyometric exercise independently will likely lead to improvements predominantly in force or velocity alone [8]. This optimization of the force–velocity relationship may further promote the recruitment of fast-twitch muscle fibers, thereby helping to maximize athletic performance (e.g., sprints, jumps, and change-of-direction speed) [8,38]. 

Furthermore, CCT may also induce hormonal (e.g., increased testosterone) [39] and structural adaptations (e.g., increased leg volume) [40], favoring the strength–power development observed through an improvement in peak torque during both knee extension and flexion, as noted in this study. Another important mechanism that may have contributed to the improvements in the CCT groups is the post-activation performance enhancement phenomenon [8,12,13], which suggests that performing a higher-load activity (e.g., heavy squat) acutely enhances the subsequent performance of a lower-load activity (e.g., jump) [18]. Indeed, a meta-analysis reported a higher magnitude of improvements in maximal strength, vertical jump, sprint, and change-of-direction ability with a CCT sequencing of exercises compared to a non-CCT approach (i.e., several heavy-load sets completed before several sets of low-load sets) [41]. 

Of note, we observed a slightly higher magnitude of improvement in the 15 m sprint (g = 1.35 vs. 0.97) and MAT (g = 3.26 vs. 0.70) for the CCT-3 group compared to the CCT-2 group, and in the SLJ (g = 1.12 vs. 0.63) and CMJA (1.61 vs. 1.06) for the CCT-2 group compared to the CCT-3 group. The greater improvements observed in MAT performance can be partially explained by the window of adaptation mechanism [42]. The baseline score for the MAT was significantly different between the CCT-3 and CCT-2 groups (Bonferroni-adjusted *t*-test *p* = < 0.001), with the CCT-3 group being comparatively slower than the CCT-2 group at baseline (mean: 6.39 s vs. 5.84 s). Therefore, the CCT-3 group had a greater opportunity to improve during the MAT. Furthermore, greater training frequency may have also allowed for greater neuromuscular adaptations and improved running efficiency (e.g., enhanced stretch–shortening cycle function). However, for the 15 m sprint, the baseline scores were not different. Therefore, the improvement in the 15 m sprint may be solely attributed to the greater training frequency, which may have led to adaptations that enhanced the utilization of the stretch–shortening cycle. Indeed, previous studies have suggested that more frequent neuromuscular stimuli may optimize motor learning [25], and thus, frequent plyometric actions in a CCT-3 program over one week may produce such adaptions. 

Additionally, the baseline score for CMJA was significantly greater for the CCT-2 group compared to the CCT-3 group (mean: 44.6 cm vs. 38.03 cm). Contradictory to previous findings observed for the MAT, here, we observed that the group (CCT-2) with a greater CMJA performance at baseline (i.e., lower body power) had a larger magnitude of improvement. The lower training frequency may have also allowed for greater recovery, thereby improving jump performance (i.e., SLJ and CMJA). However, the interpretation of results such as greater improvements in the CCT-2 group for jumps compared to greater improvements in the CCT-3 group for the l5 m sprint and MAT may be possible through electromyographic analysis during the activities. Additionally, with similar lower limb maximal strength (1-RM for squat and Romanian deadlift), a CCT program appears to be more effective in improving vertical jumps for individuals with greater explosiveness. Whether such a mechanism exists needs to be confirmed in future studies with post-activation performance enhancement assessments across individuals with similar maximal strength, but with different explosive power capabilities. Furthermore, no differences in improvements were observed between both CCT training frequencies for the30 m linear sprint, MBT, and isokinetic leg strength. Results for the isokinetic leg strength test indicated that there were no differences in the magnitude of improvement between the CCT-2 and CCT-3 groups, which is in line with previous literature that has suggested that training frequency is a less decisive moderator for lower limb strength when training load is equated [26,43,44]. 

Although the findings derived from this study may be useful for practitioners for making evidence-based decisions regarding weekly CCT frequency to optimize physical fitness improvements, potential limitations of this study should be considered when interpreting our findings. Firstly, the participants included in the study were only active male students. Therefore, the extrapolation of the findings from this study to other populations (e.g., females and trained athletes) should be made with caution. Secondly, the study was limited to a six-week duration. Although training adaptations tend to occur after six weeks, a study of longer duration should be conducted to verify the long-term effects of load-equated frequency moderators. Thirdly, although a sample size estimation was conducted, a larger sample size may be required for more robust conclusions. Fourthly, the absence of biomechanical or physiological comparisons in this study limited a more comprehensive interpretation and explanation of results. Fifthly, although we found excellent inter-assessor reliability for both the sprint and MAT measurement protocols using a stopwatch, the inclusion of electronic timing gates would be recommended in future studies to further improve precision. Lastly, the inclusion of subjective measures of effort or fatigue such as the session rating of perceived exertion or the readiness questionnaire would have offered an additional insight into the understanding of physical fitness and the related psychobiological responses to different CCT frequencies.

## 5. Conclusions

Compared to the active controls, the load-equated CCT-2 and CCT-3 programs appear to have had similar effects on most measures of physical fitness in active adult males. Of note, the CCT-3 program provided greater improvements compared to those of the CCT-2 program for the change-of-direction speed. Therefore, practitioners can use this information as a basis for designing training programs to elicit the desired physical fitness adaptations in their athletes. Moreover, for active adults with no previous CCT experience, a progressive load increment of 10% of the individual’s 1-RM every fortnight, with lower loads (e.g., 65% 1-RM) during the initial weeks for primarily anatomical adaptations. Furthermore, we did not monitor any training or test-related injuries in this study; therefore, it appears that the implementation of CCT was safe and effective for both training frequencies.

## Figures and Tables

**Table 1:** Participant demographic and anthropometric information for the two complex contrast training groups and the active control group.

	CCT-2 (n = 15)	CCT-3 (n = 15)	CG (n = 15)	*p*-Value
Age (yrs)	21.9 ± 2.3	21.1 ± 1.9	21.3 ± 1.8	0.494
Body height (cm)	177.1 ± 7.5	171.9 ± 8.2	172.5 ± 5.8	0.112
Body mass (kg)	69.4 ± 7.6	62.7 ± 7.1	67.0 ± 5.0	0.028

CCT-3—complex contrast training group, three sessions per week; CCT-2—complex contrast training group, two sessions per week; CG—control group.

**Table 2:** Absolute and relative one-repetition maximums (1 RM) of participants in the two versus three weekly frequency complex contrast training (CCT) groups.

	CCT-2	CCT-3	*p*-Value
Squat 1-RM	107.0 ± 17.6	100.3 ± 11.3	0.227
Squat relative 1-RM	1.55 ± 0.22	1.62 ± 0.23	0.414
Lunge 1-RM	63.7 ± 14.7	58.0 ± 8.6	0.208
Lunge relative 1-RM	0.92 ± 0.19	0.93 ± 0.16	0.840
Romanian deadlift 1-RM	89.0 ± 19.6	83.7 ± 12.2	0.378
Romanian deadlift relative 1-RM	1.28 ± 0.23	1.35 ± 0.22	0.421
Bench press 1-RM	73.0 ± 12.4	67.3 ± 10.8	0.193
Bench press relative 1-RM	1.05 ± 0.15	1.08 ± 0.18	0.648

**Table 3:** Protocols for complex contrast training interventions.

	High-Load Low-Velocity Exercises		Low-Load High-Velocity Exercises	
	Exercise	Repetitions per Set *	Exercise	Repetitions per Set
Weeks 1–2	Squat	15	Squat jump	6
65% 1 RM	Romanian deadlift	15	Kettlebell swing	10
	Barbell lunge	15	Barbell high knees	15 s
	Bench press	15	Plyo push-up	6
Weeks 3–4	Squat	10	Squat jump	8
75% 1 RM	Romanian deadlift	10	Kettlebell swing	10
	Barbell lunge	10	Barbell high knees	20 s
	Bench press	10	Plyo push-up	8
Weeks 5–6	Squat	6	Squat jump	10
85% 1 RM	Romanian deadlift	6	Kettlebell swing	10
	Barbell lunge	6	Barbell high knees	25 s
	Bench press	6	Plyo push-up	10

*: The number of sets was 3 for the group performing three training sessions per week. For the group performing two training sessions per week, 4 sets were performed per training session, plus an additional set performed with an equal distribution between the first and second session of the week.

**Table 4:** Statistical comparisons between the two experimental groups and the active control group according to the examined physical fitness measures.

	Complex Contrast Training Group (n = 15; 2 Sessions/Week) (CCT-2)			Complex Contrast Training Group (n = 15; 3 Sessions/Week) (CCT-3)			Active Control Group (CG) (n = 15)			Time × Group
	Pre-Test	Post-Test	*p*-Value (g) Magnitude	Pre-Test	Post-Test	*p*-Value (g) Magnitude	Pre-Test	Post-Test	*p*-Value (g) Magnitude	*p*-Value (ɳ_p_^2^)
	Mean ± Standard Deviation			Mean ± Standard Deviation			Mean ± Standard Deviation			
**Speed**										
15 m sprint (s)	2.74 ± 0.14	2.61 ± 0.12	<0.001 (0.97) Moderate	2.88 ± 0.12	2.72 ± 0.11	<0.001 (1.35) Large	2.80 ± 0.2	2.81 ± 0.19	0.697 (0.05) Trivial	<0.001 (0.52) Large
30 m sprint (s)	4.68 ± 0.23	4.53 ± 0.22	<0.001 (0.65) Moderate	4.77 ± 0.18	4.60 ± 0.20	<0.001 (0.87) Moderate	4.61 ± 0.30	4.62 ± 0.25	0.582 (0.04) Trivial	<0.001 (0.57) Large
**Muscle Power**										
Medicine ball throw	11.7 ± 1.5	12.4 ± 1.5	<0.001 (0.49) Small	10.1 ± 1.3	10.8 ± 1.4	<0.001 (0.52) Small	10.9 ± 1.4	10.9 ± 1.2	0.942 (0.00) Trivial	<0.001 (0.51) Large
Standing long jump (m)	2.4 ± 0.1	2.6 ± 0.1	<0.001 (1.12) Moderate	2.4 ± 0.2	2.5 ± 0.2	<0.001 (0.63) Small	2.3 ± 0.2	2.3 ± 0.2	0.86 (0.05) Trivial	<0.001 (0.54) Large
CMJ with arm thrust (cm)	44.5 ± 3.9	51.2 ± 4.2	<0.001 (1.61) Large	38.0 ± 4.1	43.4 ± 5.6	<0.001 (1.06) Moderate	37.5 ± 4.3	36.5 ± 3.4	0.104 (0.20) Small	<0.001 (0.70) Large
**Change-of-direction speed**										
Modified agility T-test (s)	5.84 ± 0.20	5.70 ± 0.19	<0.001 (0.70) Moderate	6.39 ± 0.26	5.32 ± 0.37	<0.001 (3.26) Very large	6.11 ± 0.32	6.14 ± 0.3	0.729 (0.09) Trivial	<0.001 (0.78) Large
**Muscle strength**										
PT knee extension (right) (N.m)	168.9 ± 23	185.3 ± 27.3	<0.001 (0.63) Small	160.8 ± 58.4	179.1 ± 59	<0.001 (0.30) Small	177.7 ± 43.1	178.9 ± 42.5	0.413 (0.03) Trivial	<0.001 (0.69) Large
PT knee extension (left) (N.m)	170.0 ± 28.1	186.3 ± 30.4	<0.001 (0.54) Small	170.3 ± 57.4	187 ± 59.2	<0.001 (0.28) Small	181.7 ± 38	182.7 ± 37.9	0.407 (0.03) Trivial	<0.001 (0.73) Large
PT knee flexion (right) (N.m)	110.1 ± 26.4	122.1 ± 27.4	<0.001 (0.43) Small	102.1 ± 25.9	112.6 ± 28.8	<0.001 (0.37) Small	102.5 ± 26.6	103.7 ± 26.1	0.251 (0.04) Trivial	<0.001 (0.63) Large
PT knee flexion (left) (N.m)	103.9 ± 20.6	115.6 ± 22.8	<0.001 (0.52) Small	100.9 ± 25.4	110.1 ± 26.1	<0.001 (0.35) Small	99.5 ± 23.2	99.9 ± 22.4	0.676 (0.02) Trivial	<0.001 (0.65) Large

Note: A re-analysis using ANCOVA was conducted with the pre-test scores as covariates for variables with significant baseline differences (i.e., medicine ball throw, CMJ with arm thrust, and modified agility T-test) and similar results were obtained (all *p* < 0.001). g, Hedges’ g; ɳ_p_^2^, partial eta squared; N.m, Newton metres; PT, peak torque; CMJ, countermovement jump.

## Data Availability

All data generated or analyzed during this study will be/are included in the published article as table(s) and figure(s). Any other data requirement can be directed to R.K.T.
